# Wear Resistance of Steels with Surface Nanocrystalline Structure Generated by Mechanical-Pulse Treatment

**DOI:** 10.1186/s11671-017-1917-z

**Published:** 2017-02-27

**Authors:** Hryhoriy Nykyforchyn, Volodymyr Kyryliv, Olha Maksymiv

**Affiliations:** Karpenko Physico-Mechanical Institute of NAS of Ukraine, 5 Naukova Str., Lviv, 79060 Ukraine

**Keywords:** Nanocrystalline structure, Wear resistance, Friction coefficient, Surface layer, Mechanical pulse treatment, Surface alloying, Carbon steel

## Abstract

The influence of the surface mechanical-pulse treatment based on high-speed friction with a rapid cooling by the technological environment on the wear resistance of medium- and high-carbon steels was considered. The treatment due to a severe plastic deformation enabled obtaining the nanocrystalline structure with a grain size of 14–40 nm. A high positive effect of this treatment was obtained not only because of metal nanocrystallization but also thanks to other factors, namely, structural-phase transformations, carbon saturation of the surface due to decomposition of the coolant and the friction coefficient decrease. Higher carbon content leads to better strengthening of the surface, and its microhardness can reach 12 GPa.

## Background

Structural-metallurgical factors which form wear resistance of steels mainly consist in choosing chemical composition of a metal and its heat treatment. The different methods of surface treatment are widely used for this goal.

From the point of high wear resistance, a special attention has been paid to steels with nanocrystalline structure (NCS) which have a complex, unique mechanical properties [1–3]. Their main advantage consists in a possibility to combine high characteristics of strength, plasticity, and brittle fracture resistance. Hence, the studies of crack growth resistance in NCS materials [4, 5] and their stability in aggressive environments [6, 7] are of a great interest. However, the complexity of such properties can be considered as the factors for improvement of wear resistance of the material. Besides, surface NCS are characterized by lower friction coefficient [8] as compared with traditional microcrystalline structure.

One of the most widely used processes for NCS production is severe plastic deformation (SPD) which allows formation of both three-dimentional and surface NCS. In the latter case it includes, namely, vibrational balls cold-work hardening [9], sand-blasting, wire brush hardening, and ultrasonic impact treatment [10, 11]. One of the methods of creating the surface NCS is mechanical-pulse treatment (MPT) which results in the formation of so-called white layer [3, 5–7]. The physics of MPT consists in the heating of the surface layers of the treated metal during high-speed friction with a rotational cylindrical tool above the phase transformation temperatures (850–1500 °C), a simultaneous thermoplastic deformation, and a subsequent rapid cooling with a speed of 10^3^–10^4^ K/s due to heat transfer from surface layers into the coolant, strengthening tool, and the treated component. It should be noted that the coolant serves not only for rapid cooling during the MPT but also for saturation of surface layers by coolant elements because of its decomposition. MPT is based on the principles of grinding and can be realized on slightly modified lathes and grinding machines. MPT enables obtaining of the surface NCS with a grain size of 12–60 nm, and microhardness reaches 7–12 GPa.

The middle, high-carbon and alloyed steels after quenching and low-temperature annealing are widely used in the industry as high wear resistant materials. The aim of this work is (a) to investigate the wear resistance of some carbon steels with a different chemical composition with the surface NCS formed by MPT; (b) to compare the wear resistance of certain steels after different treatments, quenching with following low-temperature tempering and after MPT strengthening.

## Methods

The studied steels were 35 (0.35C), 45 (0.45C), U8 (0.8C), 40Kh (0.4C-1Cr), and a ball-bearing steel ShKh15 (1C-0.3Si-0.3Mn-0.3Ni). All investigated steels were treated by MPT after normalizing and tempering. The steels 40Kh and ShKh15 were quenched and tempered at the temperature of 200 °C additionally to achieve the hardness 52–54 HRC (5.9–6.1 GPa) and 60-62 HRC (7.7–8.4 GPa), correspondingly.

MPT was carried out using the strengthening tool made of a titanium alloy VT6 (Ti-6Al-4V) with the following treatment regimes: a tool rotating at a velocity of 60 m/s, a treated specimen rotating at a velocity of 0.04 m/s, a longitudinal feed of the tool along the component axis of 0.8 mm/rev, and a depth of run that was equal to 0.35 mm (the value characterized a pressing force of the tool on the treated component). The special technological medium for carburizing [12] with additives of a low-molecular polyethylene as a carbon content diffusing substance was used as a coolant.

Phase analyses of the surface layers after MPT were carried out with the CuK_α_-radiation method (*U* = 30 kV, *I* = 20 mA) with a step of 0.05° and an exposition of 4 s. The diffractograms were post processed using the software Powder Cell. The X-ray pictures were analyzed using the JCPDS-ASTM index [13].

**Fig. 1:**
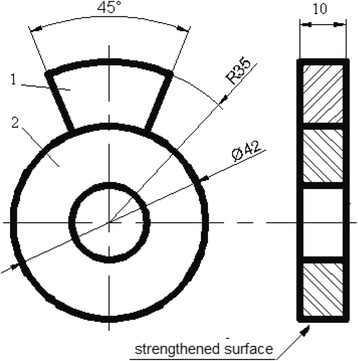
The scheme of the wear resistance study. (*1*) insert. (*2*) ring

Wear resistance of the steels were studied according to the scheme (Fig. 1): ring (specimen)–insert (counter body). The investigations were carried out in GOST TAP-30 oil-abrasive medium with addition of fine quartz sand (0.1 mas.%) with a grain size of up to 40 μm. The ring specimens with a diameter of 42 mm and a thickness of 10 mm treated with MPT cylindrical surface were used. The specimen rotation provides the linear sliding velocity of 0.9 m/s. The material of the insert was the steel ShKh15 in as-received state, the insert-specific pressure on the ring was 1 MPa. The wear parameter was assumed as the weight loss with time, which was established gravimetrically with a precision of ±4 mg.

The same scheme was used to establish the friction coefficient in air and in oil without addition of quartz sand. The insert-specific pressure on the ring varied between 2.0 and 6.0 MPa.

## Results and Discussion

It was established by the X-ray analysis that MPT generated ferrite-austenite steels (35, 45, 40Kh steels) and ferrite-cementite (U8, ShKh15 steels) structure. The grain size in the surface layers was 14–40 nm.

**Fig. 2:**
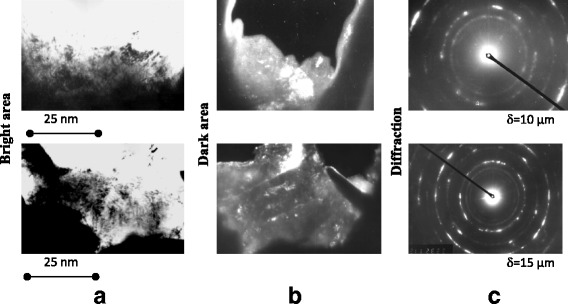
Electron microscope images and diffraction patterns of steel 45 structures. Structures **a** *light field*, **b** *dark field*, and **c** diffraction patterns of NCS steel 45 surface layers were made at a different depth δ after MPT

The electron microscopy images of the NCS that strengthened layer of the steel 45 from a depth of 10 and 15 μm are presented in Fig. 2a, b. The grains are strongly fragmented and their boundaries are distorted, which indicates great stresses there. The dislocation clews are observed on the grain boundaries. Such diffraction patterns (Fig. 2c) are typical for NCS; the grains are strongly disorientated (the grain boundary angles achieves 20° and above). The grain size on the surface is about 20 nm. In addition, the presence of oxides FeO and Fe_3_O_4_ on the metal surface are detected by the X-ray analysis.

**Fig. 3:**
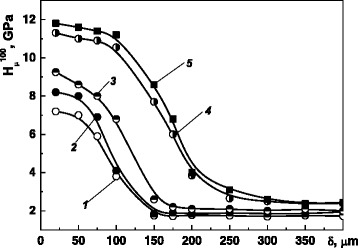
Microhardness distribution in a depth from surface for the different steels after MPT. Microhardness was measured on the following steels: (1) 35; (2) 45; (3) 40Kh; (4) U8; (5) ShKh15

Microhardness H_μ_ of the surface layer of steels with the surface NCS (Fig. 3) is 4-6 times higher compared to that of the untreated material. The depth of the strengthened layer is about 200 μm. This is a result of the nanostructurization of the surface as well as other important factors: the saturation of the surface layer with carbon from a technological medium [12]; the structural-phase transformations as a result of thermal-mechanical treatment (like quenching); and the strain hardening of the material.

It should be noted that the MPT effect became more pronounced with increase of carbon content in the steels. In general, the influence of carbon amount on hardness of untreated metal layers in the depth of specimen is negligible. However, the surface layers of 200 μm thick after MPT of different steels have similar dependences of the microhardness from the depth of strengthening creating two zones: 1–3 curves for steels with carbon content in a range of 0.35–0.45% and curves 4–5 0.8–1.0% (Fig. 3).

**Fig. 4:**
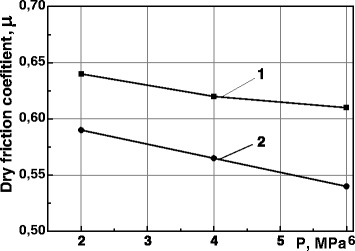
Dependence of the dry friction coefficient *μ* of steel 35 from a specific load. The friction coefficient was determined in the air in the friction pair steel 35 (specimen)–steel ShKh15 (counter body). (*1*) as-received state of steel 35. (*2*) Steel 35 after MPT

The friction coefficient *μ* is slightly decreased in air (dry friction) with the increase of the insert specific load on a specimen (Fig. 4), but the regularity of *μ* decreased after MPT is remained. This effect could be explained only by a higher hardness of the material. However, it should be noted that a high microhardness of the NCS layer is concerned with the localization degree of valence electrons since high pressure activates s-d or s-d-f electron transition [14]. From another point of view, it could be explained by a change of the contribution of electrons of *d* orbitals in metallic bond which results in friction coefficient decrease: the stronger the atomic bond is inside the metal, the weaker the bond is on the interfaces [15].

Comparison of the friction coefficients of quenched and strengthened by MPT specimens from steel 45 in oil showed significant difference (more than in 3 times) in *μ* values (Table 1). In other words, the influence of the MPT on *μ* is stronger in the conditions of oil friction than in air. It is obvious that such sharp decrease in friction coefficient leads to wear resistance improvement.

**Table 1:** Dependence of the friction coefficient in the pair steel 45 (specimen)–steel ShKh15 (counter body) in oil on the kind of treatment

Kind of treatment	*μ*
Quenching and tempering	0.19
MPT	0.05

**Fig. 5:**
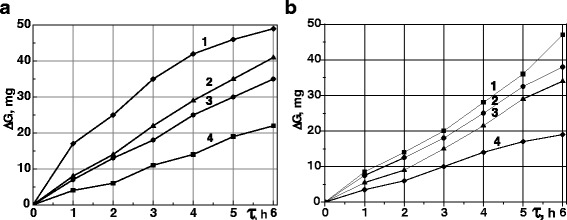
Kinetics of wear of rings **a** and inserts **b** from the steels 40Kh and ShKh15. The investigations were realized in conditions of oil-abrasive friction on the steels 40Kh (*1*, *2*) and ShKh15 (*3*, *4*) after quenching and tempering (*1*, *3*) and after MPT (*2*, *4*)

The wear resistance of steels 40Kh and ShKh15 after MPT is higher than the same steels after quenching and low tempering which are confirmed by wear resistance investigations (Fig. 5a). It should be noted that formed during MPT oxides FeO and Fe_3_O_4_, being secondary structures could also decrease wear by the way of relieve running-in of the friction pairs [16].

**Fig. 6:**
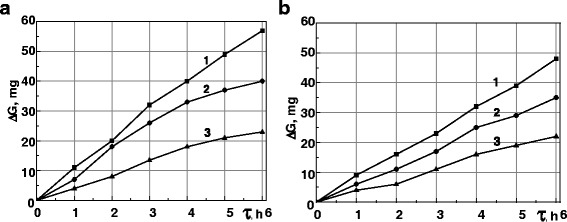
Kinetics of wear of rings **a** and inserts **b** from the steels 35, 45, and U8. The investigations were realized in conditions of oil-abrasive friction on the steels 35 (*1*), 45 (*2*), and U8 (*3*) after MPT

The wear resistance of the surface NCS rises with an increase of carbon content in the steel (Fig. 6a), and it is in good agreement with the dependence of microhardness on carbon content (Fig. 3): higher carbon content provides higher microhardness and, correspondingly, higher wear resistance in the friction pairs.

The positive effect of the rings MPT strengthening on the wear resistance of untreated inserts (Figs. 5b and 6b) could be explained by a decrease in the friction coefficient in the specimen—counter body friction pair. Hence, it confirms the importance of *μ* decreasing for wear resistance increase of the steels after MPT.

A rise of the wear resistance of the steel surface NCS could be expected in the case of the hydrogen saturation of the metal, which frequently happens in real service conditions. As it was showed before [6, 17], the NCS on steels obtained by MPT can weaken the hydrogen penetration in the metal and can increase the resistance of the hydrogen embrittlement. It leads to reduction of wear intensity, at least, by the chipping mechanism.

## Conclusions

1. The influence of the surface mechanical-pulse treatment based on high-speed friction and cooling by technological medium on the wear resistance of the medium- and high-carbon steels is considered.
2. The grain sizes, determined by X-ray analysis, were in the range of 14–40 nm. The electron microscopy analysis confirmed these results and indicated strongly distorted grain boundaries and dislocation clews. These diffraction patterns are typical for a great disorientation of the grains.
3. The effect of the surface mechanical-pulse strengthening is a result of some factors: the surface nanostructurization, structural-phase transformations, carbon surface saturation from destructed technological medium during the treatment. The carbon content increment provides stronger effect of strengthening of the steel. The microhardness reaches 7-12 GPa for the steels with carbon content of 0.35–1%.
4. Mechanical-pulse treatment improves significantly the wear resistance of the steels in comparison with the quenching and low temperature tempering ones.
5. The significant decrease of friction coefficient as a result of the mechanical-pulse treatment of surface leads to an increase of the wear resistance of both strengthened specimen and unhardened counter body.
